# Reduced Dental Plaque Formation in Dogs Drinking a Solution Containing Natural Antimicrobial Herbal Enzymes and Organic Matcha Green Tea

**DOI:** 10.1155/2016/2183623

**Published:** 2016-10-27

**Authors:** Michael I. Lindinger

**Affiliations:** Research and Development, The Nutraceutical Alliance, Campbellville, ON, Canada L0P 1B0

## Abstract

The results of an exploratory, multicenter clinical study confirmed the hypothesis that a novel, natural, and safe oral care product (OCP) reduced the rate of plaque formation on teeth of dogs consuming the OCP (antimicrobial plant-derived enzymes, organic matcha green tea, cultured dextrose, sodium bicarbonate, and ascorbic acid) compared to controls. Healthy dogs without periodontitis, of varying breeds, sex, and age, were recruited and enrolled, using nonrandomized stratification methods, into a control and treatment groups. Treatment group dogs drank only water into which OCP was suspended, for 28 days. Control group dogs drank their normal household water. On day 0 all teeth were cleaned by a veterinarian and gingivitis was assessed. On days 14, 21, and 28 plaque index, plaque thickness, gingivitis, freshness of breath, and general health were assessed. Over the 28 days of study, dogs on the OCP had significant reduction in plaque index and plaque thickness compared to controls. By day 14 OCP reduced plaque formation by 37%; the 28-day reduction in plaque index and coverage averaged 22% with no measurable gingivitis or calculus.* Conclusion*. Using the OCP attenuated dental plaque formation when consumed as normal drinking water and in the absence of other modes of oral care.

## 1. Introduction

Periodontal disease is the most prevalent of all diseases in dogs and cats and a primary cause of health-related ailments [1], with up to 80% of pets experiencing periodontal disease by the age of 3 years. Global cost of treating the disease is high, running into billions of dollars (US) annually [2]. Nutraceutical and natural products are increasingly being used and developed for improving many aspects of health and wellbeing in people and other mammals [3–6]. At present, there are few nutraceutical products aimed at supporting dental and oral health [5], and there is a paucity of research demonstrating efficacy and safety of these in nonhuman target species. This paper reports the effects of a nutraceutical product on indices of oral health in dogs living in their owners' homes and assessed through regular visits to their veterinary clinic.

Pet owners are often slow to accept that pets have similar oral care concerns as humans and that regular oral hygiene is of importance to their pet's overall health and wellbeing. Even when this is recognized, owners are often reluctant to perform routine oral care for their pets. This has led to the development of an industry targeted at oral care that has developed a wide range of products such as chew toys, tooth brushes, oral foams and gels, sealants, special feeds, and drinking water additives. These are only effective when used, and consistent use of products continues to be a concern for pet owners. With this in mind, the present study reports on the testing of an oral care product that is used as regularly and as often as dogs and cats need to drink.

The proprietary OCP that was tested was formulated using naturally occurring and beneficial compounds that have been shown to (1) inhibit the proliferation of orally-occurring bacteria [6–8]; (2) have antioxidant and anti-inflammatory potential [9, 10]; and (3) have breath freshening and tooth whitening properties [3, 11]. It was hypothesized that, compared to control dogs, dogs drinking water into which this OCP was suspended would exhibit a reduced rate of plaque formation, reduced plaque thickness, reduced calculus, and an absence of or minimal gingivitis. Dogs drank water containing the OCP for 28 days, compliance was good, and no adverse events were reported by owners or veterinarians.

## 2. Materials and Methods

### 2.1. Animals and Recruitment

Dogs for inclusion in the study were recruited through one large veterinary clinic in the United Kingdom and three smaller veterinary clinics in Canada. Veterinarians led owners through the informed consent process, and obtained signatures of agreement from all owners of dogs recruited into the study. Inclusion criteria required that these were healthy dogs with no medical concerns including absence of periodontitis. The veterinarians completed a recruitment record indicating the parameters listed in Table 1. All dogs lived in their owners' homes during the course of study and were instructed to report to their veterinary clinic at designated time points. The dog care, treatment, and handling procedures were approached by the Animal Care Committee of the Nutraceutical Alliance, in conformity with the guidelines of the Canadian Council on Animal Care.

Each owner signed an informed consent for inclusion of their dog in the study and was given oral and written instructions regarding oral care while in the study. Specifically, owners were instructed to not give chew toys or any dental/oral care type products to their dogs for the next 28 days. Owners of dogs in the treatment trial were further instructed to allow only water into which the OCP was suspended and, if needed, to gradually increase the strength of the solution to that recommended over a period of 3–7 days. The treatment group dogs were not to have access to regular water at any time, including outdoor walks, and if a drink was needed then the owner was to bring the OCP-treated water.

The veterinarian assigned dogs to either the control group or treatment group using nonrandomized stratification methods, with instructions to achieve balance in each group with respect to breed, age, sex, type of food eaten, and weight. However, of the dogs used for data analysis there were 12 in the control group and 17 in the treatment group. The care and use of the dogs conformed to the standards of the Canadian Council of Animal Care.

### 2.2. Assessment

Approximately one week after recruitment, each dog visited the veterinary clinic for assessment and complete dental cleaning. Plaque and calculus indices, thickness, and breath freshness were assessed prior to cleaning (see below). All plaque and calculus were removed using cleaning and hand scaling techniques with no or minimal sedation. Supra- and subgingival scaling of teeth surfaces were performed after periodontal probing and charting. None of the dogs recruited into the trial had visual evidence of caries and radiographs were not performed. At the completion of cleaning and polishing of teeth, gingivitis was assessed using a modified Silness-Löe gingivitis index [13] as follows.

Criteria for Assessing Gingivitis [14, 15] are

 0: absence of inflammation—normal gingiva,
 1: mild inflammation, slight change in color, slight edema, and no or mild bleeding on probing,
 2: moderate inflammation—the gingiva is red and swollen and bleeds on gentle probing of the sulcus,
 3: severe inflammation—the gingiva is red or reddish-blue, the gingival margin is swollen, and there is a tendency to spontaneous hemorrhage or profuse hemorrhage on probing and/or ulcerations along the gingival margin.

**Figure 1:**
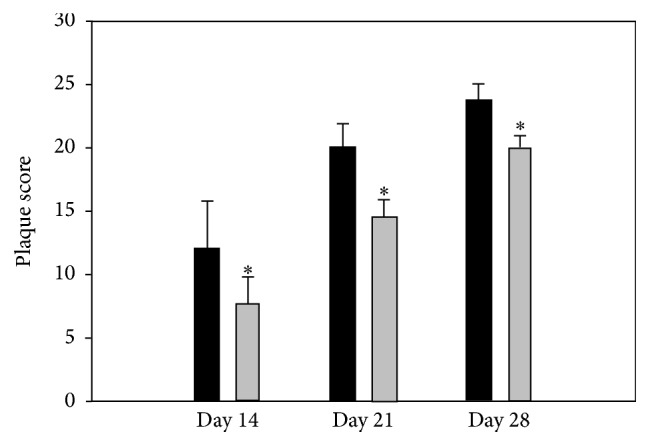
Plaque score (the sum of plaque index and plaque thickness on all assessed teeth in the mouth) in dogs in the control trial and in dogs using the oral care product (OCP). Black bars denote control dogs and grey bars denote OCP dogs. There was a significant increase in plaque score within group by each assessment day. Numbers of dogs (control, treatment): day 14: 6, 11; day 21: 9, 14; day 28: 11, 15. *∗* indicates significant difference between treatments (*p* ≤ 0.05).

Owners returned to the veterinary clinic with their dog(s) at 14, 21, and 28 days after cleaning. Not all dogs returned for days 14 and 21, resulting in different numbers of scores in the database for each of the three assessment days (see Figure 1). The gums and teeth of each dog were assessed for gingivitis (see “*criteria for assessing gingivitis [14, 15]*” in Section 2.2) and plaque (Table 2) using an accepted scoring system [12, 15, 16]. Teeth are identified anatomically where I3 is the third incisor, C is the canine, P3 is the third premolar, P4 is the fourth premolar, and M1 is the first molar. Scoring was consistently performed on the same side, with all dogs but two scored on the right side of the mouth. Gingivitis was scored lingually and facially. Plaque coverage and thickness were visualized using a handheld ultraviolet light beam, and thickness was also assessed with blunt probing. Gingivitis was assessed with blunt probing. Care was taken on days 14 and 21 to not remove existing plaque. The veterinary technician who performed the scoring was trained in the procedures, blinded as to whether the dog was control or treatment, and manually recorded the results onto paper record pages. These were scanned into digital format and the digital records archived by the principal investigator. A plaque score for each dog for each assessment day was calculated by taking the sum of the individual plaque index and thickness scores. Breath freshness was assessed subjectively and recorded as good or as mild halitosis; no dogs exhibited more than mild halitosis at the time of assessment.

### 2.3. Test Material

The OCP consisted of a fine greenish-colored powder that contained organic matcha green tea, sodium bicarbonate, cultured dextrose, a proprietary blend of antimicrobial plant enzymes, and ascorbic acid. The exact composition is maintained as a trade secret. Each of the ingredients present in the product has either been approved as generally recognized as safe (GRAS) or previously approved for use in human and animal feeds or in human food processing. The recommended inclusion rate was 3.6 grams per liter of drinking water, with dogs consuming water containing 0.9 g/L (*n* = 2), 1.8 g/L (*n* = 5), and 3.6 g/L (*n* = 9).

### 2.4. Statistics

Challenges associated with performing this study in an owner-friendly manner resulted in some dogs not being assessed at every time point and in some case required the need to eliminate some dogs from the final data analysis. Reasons for elimination included owner intervention with respect to oral hygiene (dogs for each group showing consistent 0 scores for plaque on days 14 and 21), regular access to normal drinking water by dogs in the treatment group, and failure to maintain the dog on the OCP.

Data were initially assessed by 3-way ANOVA with respect to treatment, day, and tooth to ascertain if there were overall differences. When a significant *F*-ratio was obtained the data were further assessed using a 2-way repeated measures ANOVA for each tooth with respect to treatment and day. When a significant *F*-ratio was obtained the Holm-Sidak post hoc test was performed. Significance was accepted at *p* ≤ 0.05.

## 3. Results

No adverse events for any of the dogs were reported by the veterinarians. Owners of two dogs reported refusal to drink water into which the OCP was suspended, and these are therefore not included in the data presented. For dogs receiving the OCP, 10 dogs received the OCP at the recommended dosage, 5 dogs at 1/2 recommended dose, and 2 dogs at 1/4 recommended dose. The reason for the lower doses was a dog aversion to the full-dose OCP-treated drinking water; owners were instructed to continue as the highest dose for which the dog showed no aversion. Statistical analysis revealed no difference in both plaque parameters between dosages, so dogs receiving the OCP for the entire trial duration were treated as a single group.

Compared to control dogs, average plaque score at each day of assessment was significantly reduced in dogs that received the OCP in their drinking water on a daily basis (Figure 1). In the first 2 weeks, the OCP reduced plaque formation by 37% on average; the 28-day reduction in plaque index and coverage averaged 22%. The reduction in plaque score was due to both reduced tooth plaque coverage and plaque thickness.

The plaque index was used as the metric for plaque coverage. The development of plaque coverage was similar amongst control teeth, ranging between 0.5 and 1 during the first 14 days (Table 3). Plaque thickness (Table 4) was in close agreement with the observations on plaque index. Overall, in both groups, there was a significant and progressive increase in plaque coverage and thickness over time, although some teeth exhibited less accumulation than others. Teeth with the greatest rate of plaque formation were the canine teeth and first molar teeth. Dogs that received the OCP routinely in their drinking water, compared to control dogs, showed a significant reduction in plaque index and thickness on the mandibular and maxillary canine and M1 teeth on day 28.

For gingivitis index there were no differences between treatments, no differences between teeth and no differences between facial and lingual tooth surfaces. Gingivitis index, collapsed by tooth, was the highest on day 0 and likely reflects the cleaning procedures just performed (Table 5). Gingivitis index was low on all subsequent assessment days compared to day 0, with no differences between days 14, 21, and 28.

There were no differences in subjective assessment of breath freshness between the two treatment groups nor between assessment days 14, 21, and 28.

## 4. Discussion

This is one of first published studies examining the ability of a nutraceutical product on oral health in dogs. By design the present study was performed using dogs free ranging in their owner's home, as opposed to using purpose-bred research dogs studied in a research environment. While this design results in a loss of internal control (variation in the age, breed, habits of the dogs, environment including consistency of drinking water, and dropouts due to inability of owners to abide by the study protocol), it does produce results that are more widely applicable to the target population in real life conditions. It was observed that, compared to control dogs, statistically significant reductions in plaque formation occurred in free ranging dogs receiving the oral care product within a 2-week period and that the reductions persisted for the study duration.

Gawor et al. [5] reported on the effects of a seaweed-containing product to improve oral health in dogs in a clinical trial. It is not possible to directly compare results between the present study and Gawor et al.'s [5] because they did not report values for plaque coverage and plaque thickness. Their “oral health index” was a score based on assessment of mandibular lymph nodes, presence of dental lesions, and degree of plaque and calculus coverage. Similar to the present study, they showed a significant reduction in oral health index associated with nutraceutical intervention over 42 days of study.

A general observation was that dogs in both treatment groups tended to exhibit plaque index and gingivitis index scores consistent with those of previously published studies. Hennet's [14] review reported plaque index scores at 4 weeks after cleaning ranging between 0.8 and 2.4 and gingivitis index ranging between 0.5 and 2.1. A numerically comparable scoring system was used by Gawor et al. [5] who reported a doubling of “oral health index” between days 14 and 28. The reasons for a seemingly high degree of variation amongst studies are unknown but may reflect preexisting level of oral hygiene, type and frequency of feed, quality of the household drinking water, frequency of drinking, and nuances in applying the scoring system.

The degree of variability was higher in the present study than anticipated. The main factors likely contributing to this are different numbers of dogs at each time point within a treatment group over time and the use of dogs in free living conditions. This variability likely accounts for the apparently higher degree of efficacy at day 14 than at day 28. Statistically, the OCP product did not exhibit diminishing efficacy from days 14 to 28, and the results reflect, at least in part, the different numbers of dogs at day 14 versus day 28.

There are several drinkable oral care products on the market, many of which have zinc gluconate as antimicrobial and other chemicals (i.e., chlorhexidine) that serve to enhance palatability and freshen breath. Use of some of these products raises their own health concerns. Excess dietary zinc, as may occur from drinking adequate amounts of zinc gluconate-containing products, may lead to dietary zinc overload (associated with neutropenia, decreased white cells, sideroblastic anemia, and digestive disorders) when used over a lifetime [17–19]. Additionally, very few products are supported with published research, raising concerns about veracity of claims and product safety.

The oral care product tested in the present study contained organic matcha green tea, a blend of antimicrobial plant enzymes, cultured dextrose, and sodium bicarbonate. A small amount of ascorbic acid is used to prevent discolouration (oxidation) of the tea when the product is introduced into the drinking water. Matcha is a fine-ground, powdered, high-quality green tea made using shade-grown tea buds (leaves). The shading slows down plant growth, stimulates an increase in chlorophyll, and causes the production of amino acids, in particular L-theanine which is GRAS [20] and antioxidant catechins.

Matcha is found in numerous health food products. The health benefits of matcha green tea may be attributed to the fact that the whole tea leaf is ingested, as opposed to just the steeped water in the case of “bagged” green teas. The concentration of the antioxidant epigallocatechin gallate (EGCG) available from matcha is at least three times greater than the amount of EGCG available from other green teas preparations. Matcha, by weight, delivers a much higher potency of catechins, chlorophyll, and other antioxidants. Teas, and especially matcha, have been shown to have high antibacterial, antitoxin, antiviral, and antifungal activities [4, 8]. Green tea is also receiving attention as a nutraceutical for supporting improved oral health [10, 21–23]. It is an excellent source of polyphenol antioxidants such as epigallocatechin 3 gallate and epicatechin 3 gallate. The antioxidant and antimicrobial activities of these catechins are beneficial in the treatment of periodontal disease and provide protection against bacterial induced dental caries and halitosis [3, 7, 9]. Furthermore, the efficacy of green tea catechins has been shown to be equal to that of chlorhexidine gluconate, a commonly used mouthwash ingredient in pet and human oral care products [6].

A potential concern of dogs consuming water into which matcha green tea has been suspended relates to the intake of methylxanthines. The trimethylxanthine caffeine was present in this blend of matcha at a concentration of 23 mg/g of matcha, and the dimethylxanthine theophylline was not detectable. Based on a 20 kg dog drinking on average 1 L of water per day, the resultant daily intake of caffeine by a dog drinking water into which the OCP was suspended would be 1.3 mg/kg body mass. This dosage is less than 1% of the LD50 for dogs and cats (median of 140 mg/kg [24, 25]) and is highly unlikely to exert untoward sympathomimetic effects in these animals.

Antimicrobial enzymes within the OCP function to inhibit the growth and proliferation of biofilm- [26, 27], plaque-, and calculus-forming bacteria [28–31] and some additionally have anti-inflammatory activity [32]. They also function to hydrolyze food starches and proteins retained in the mouth following meals, and others function to destroy bacterial cells walls. Each of these enzymes is either GRAS for use as a food additive, is on Health Canada's List of Permitted Food Additives [33], or has been approved as safe for use in the food processing industry.

Cultured dextrose is produced by the fermentation of dextrose by probiotic bacteria such as* Propionibacterium freudenreichii* and* Lactococcus lactis* that naturally produce a largely undefined mixture of fermentation metabolites that prevent microbial growth and spoilage in foods. Predominant amongst these metabolites are butyric, propionic, and lactic acids as well as small peptides. In the FDA Agency Response Letter GRAS Notice number GRN 000128, [34] it is stated that dextrose cultured with* Propionibacterium freudenreichii* subsp.* shermanii* is GRAS, through scientific procedures, for use as an antimicrobial agent in a variety of foods at a maximum level of 2% (weight/weight) in the finished product.

Sodium bicarbonate is present in the OCP to briefly raise oral pH, which impairs growth of plaque-forming bacteria [11], for its teeth-whitening properties [35], for freshening the breath [36], and adding to the favourable taste [37]. Sodium bicarbonate is an inorganic salt that has a long history of use as a food ingredient and, more recently, as a health food supplement. It is considered to be very safe for human use, with a human oral LD50 > 4,000 mg/kg bw [38, 39]. It is on the list of permitted food additives in Canada and the US and is a common ingredient found in many medicines and drug formulations. Sodium bicarbonate is GRAS as a chemical ingredient in food (21 CFR §184.173, [39]).

A number of limitations of the study deem that this research is exploratory in nature. Ideally, conformity of dogs between groups would be identical, compliance of dogs and owners would be 100%, the numbers of dogs in each group would be greater, and the procedures would benefit from being performed in a control laboratory setting. Confounds present in this study include different numbers of dogs in control and ACP groups, although they were similar in the characteristics identified. Physical activity and related eating and drinking behaviors were not assessed, and these could also contribute to differences in responses both within and between treatments.

## 5. Conclusion

In summary, the present exploratory, clinical study demonstrated that drinking water containing an oral care product (OCP), composed of naturally occurring and safe ingredients, attenuated plaque formation compared to control dogs. In the first 2 weeks, the OCP reduced plaque formation by 37% on average; the 28-day reduction in plaque index and coverage averaged 22%. Furthermore, no measurable calculus was formed (in both OCP and control dogs), gingivitis was absent, and fresh breath was maintained throughout the trial period. The product was easily accepted by most dogs recruited into the study, indicating good palatability. No adverse events were reported.

## Figures and Tables

**Table 1:** Dogs recruited into the study.

Location	ID	Breed	Age	Sex	Weight (kg)	Food type	Group
Canada	T-1	BoT	9 y 3 m	M	9.8	D	OCP
	T-2	YT	6 y 4 m	M	2.4	D	Control
	T-3	Tm	5 y 2 m	F	4.7	D	OCP
	T-4	JR	3 y 4 m	M	6.7	D	Control
	T-5	M	8 y 9 m	M	4.7	D	OCP
	T-6	M	12 y 8 m	F	4.9	D	OCP
	T-7	MT	9 y 2 m	M	7.1	D	OCP
	T-8	MS	7 y 4 m	F	8.2	D	OCP

United Kingdom	1	BT	15 y	M	8.25	D	OCP
	2 Not used	SBT	1 y	F	16.3	D	OCP
	3	SBT	10 y 10 m	M	19.5	D	Control
	4	Bull	12 y 1 m	M	22	D & W	OCP
	5	BT	2 y 2 m	M	8.75	D	Control
	6	BC	10 y 6 m	F	19.1	D	OCP
	7	JR	3 y	F	5.3	D	OCP
	8	B	4 y	F	10.4	D & W	Control
	9	SxC	10 y	M	17.5	D & W	Control
	10	CS	3 y	F	14.1	D & W	OCP
	11	BC	4 y 6 m	F	19.7	D & W	Control
	12 Refusal	JR	2 y 6 m	F	5.9	D	OCP
	13	Lab	8 y 1 m	M	29.5	D	OCP
	14	GS	2 y 1 m	M	59	D	Control
	15	MP	5 y 9 m	M	5	D & W	Control
	16	JR	3 y	M	6.5	D & W	OCP
	17	MP	4 y 8 m	F	4.5	D & W	Control
	18	BC	11 y	F	21.9	D & W	OCP
	19	C	3 y 10 m	M	13.4	D	OCP
	20	MS	6 y 4 m	M	8.9	D	Control
	21	MS	4 y 6 m	M	10.2	D	Control
	22	JR	6 y 8 m	M	8.2	D & W	OCP
	23	JR	6 y 8 m	M	10.6	D & W	OCP

B: Bedlington; BC: Border Collie; BoT: Border Terrier; BT: Boston Terrier; Bull: Bull Terrier; C: Cockerpoo; CS: Cocker Spaniel; GS: German Shepherd; Lab: Labrador; JR: Jack Russel; M: Maltese; MP: Miniature Pinscher; MS: Miniature Schnauzer; MT: Manchester Terrier; SBT: Staffordshire Bull Terrier; SxC: Springer-Cocker Cross; Tm: Terrier Mix; YT: Yorkshire Terrier. OCP: oral care product; D: dry food; W: wet food.

**Table 2:** Criteria for scoring plaque, where plaque is defined as “soft debris” and is visualized using ultraviolet illumination (modified Logan and Boyce as described by Hennet et al. [12]).

Score	Coverage	Thickness/intensity
0	None observable	0: none
1	1–24% coverage	1: light = pink to light red
2	25–49% coverage	2: medium = red
3	50–74% coverage	3: heavy = dark red
4	75–100% coverage	

**Table 3:** Plaque index (coverage scores) on selected teeth in the lower (mandibular) and upper (maxillary) jaws of control and treatment dogs. Mandibular I3 was not part of the assessment.

Tooth	Day	Lower Jaw		Upper Jaw	
		Control	OCP	Control	OCP
Canine	14	0.75 ± 0.3	0.46 ± 0.16^*∗*^	0.75 ± 0.25	0.46 ± 0.16^*∗*^
	21	1.4 ± 0.2^∧^	0.56 ± 0.18^*∗*∧^	1.6 ± 0.2^∧^	0.63 ± 0.18^*∗*∧^
	28	1.5 ± 0.2	1.1 ± 0.1^*∗*∧^	2.0 ± 0.2	1.4 ± 0.2^*∗*∧^

M1	14	1.0 ± 0.00	0.46 ± 0.16^*∗*^	0.75 ± 0.25	0.46 ± 0.16^*∗*^
	21	1.4 ± 0.2^∧^	0.89 ± 0.11^*∗*∧^	1.6 ± 0.2^∧^	1.0 ± 0.00^*∗*∧^
	28	1.6 ± 0.2	1.1 ± 0.1^*∗*∧^	1.7 ± 0.2	1.2 ± 0.1^*∗*^

P3	14	0.50 ± 0.29	0.36 ± 0.15	0.50 ± 0.29	0.46 ± 0.16
	21	1.0 ± 0.00^∧^	0.67 ± 0.17^*∗*∧^	1.0 ± 0.00^∧^	1.0 ± 0.00^∧^
	28	1.0 ± 0.00	0.92 ± 0.08^∧^	1.3 ± 0.2	1.2 ± 0.2

P4	14	0.50 ± 0.29	0.36 ± 0.15	0.50 ± 0.29	0.50 ± 0.16
	21	1.0 ± 0.00^∧^	0.67 ± 0.17^*∗*∧^	1.0 ± 0.00^∧^	1.2 ± 0.1^∧^
	28	1.0 ± 0.00	1.0 ± 0.00^∧^	1.0 ± 0.00	1.2 ± 0.2

I3	14	—	—	1.0 ± 0.00	0.28 ± 0.14^*∗*^
	21	—	—	1.2 ± 0.2	0.75 ± 0.16^*∗*∧^
	28	—	—	1.4 ± 0.2	1.3 ± 0.2^∧^

Values are mean ± se. Numbers of dogs (control, treatment): day 14: 9, 16; day 21: 9, 15; Day 28: 11, 17.

*∗* indicates significant difference (*p* < 0.05).

∧ indicates significant difference (*p* < 0.05) compared to the preceding time point.

**Table 4:** Plaque thickness scores on selected teeth in the lower (mandible) and upper (maxilla) jaws of control and treatment dogs. Mandibular I3 was not part of the assessment.

Tooth	Day	Lower Jaw		Upper Jaw	
		Control	OCP	Control	OCP
Canine	14	0.75 ± 0.25	0.46 ± 0.16^*∗*^	0.75 ± 0.25	0.46 ± 0.16^*∗*^
	21	1.4 ± 0.2^∧^	0.56 ± 0.18^*∗*^	1.4 ± 0.2^∧^	0.63 ± 0.18^*∗*^
	28	1.3 ± 0.2	0.92 ± 0.08^*∗*∧^	1.8 ± 0.3^∧^	1.2 ± 0.1^*∗*∧^

M1	14	1.0 ± 0.00	0.46 ± 0.16^*∗*^	0.75 ± 0.25	0.46 ± 0.16^*∗*^
	21	1.4 ± 0.2^∧^	0.89 ± 0.11^*∗*∧^	1.6 ± 0.2^∧^	1.0 ± 0.00^*∗*∧^
	28	1.6 ± 0.2	0.92 ± 0.08^*∗*^	1.7 ± 0.2	1.1 ± 0.1^*∗*^

P3	14	0.50 ± 0.29	0.36 ± 0.15	0.50 ± 0.29	0.46 ± 0.16
	21	1.0 ± 0.00^∧^	0.67 ± 0.17^*∗*∧^	1.0 ± 0.00^∧^	1.0 ± 0.00^∧^
	28	1.0 ± 0.00	0.92 ± 0.08^∧^	1.2 ± 0.2	1.1 ± 0.1

P4	14	0.50 ± 0.29	0.36 ± 0.15	0.50 ± 0.29	0.55 ± 0.16
	21	1.0 ± 0.00^∧^	0.67 ± 0.17^*∗*∧^	1.0 ± 0.00^∧^	1.2 ± 0.1^∧^
	28	1.0 ± 0.00	1.0 ± 0.00^∧^	1.0 ± 0.00	1.1 ± 0.2

I3	14	—	—	1.0 ± 0.00	0.28 ± 0.14^*∗*^
	21	—	—	1.0 ± 0.00	0.75 ± 0.16^*∗*∧^
	28	—	—	1.1 ± 0.1	1.1 ± 0.2^∧^

Values are mean ± se. Numbers of dogs (control, treatment): day 14: 9, 16; day 21: 9, 15; day 28: 11, 17.

*∗* indicates significant difference (*p* < 0.05).

∧ indicates significant difference (*p* < 0.05) compared to the preceding point.

**Table 5:** Gingivitis index for facial and lingual surfaces of all teeth assessed (collapsed by tooth) of control and treatment (OCP) dogs. Note that the gingivitis index on day 0 represents effects of cleaning on the gingiva. No cleaning was performed prior to gingival assessments on days 14, 21, and 28. There were no differences between treatments or between days 14, 21, and 28.

Day	Gingivitis index, lingual		Gingivitis index, facial	
	Control	OCP	Control	OCP
0	0.68 ± 0.03	0.78 ± 0.04	0.70 ± 0.04	0.73 ± 0.04
14	0.06 ± 0.03	0.06 ± 0.04	0.02 ± 0.04	0.02 ± 0.04
21	0.00 ± 0.07	0.00 ± 0.00	0.00 ± 0.04	0.00 ± 0.04
28	0.07 ± 0.04	0.10 ± 0.04	0.00 ± 0.04	0.00 ± 0.00

Values are mean ± se. Numbers of dogs (control, treatment): day 14: 9, 16; day 21: 9, 15; day 28: 11, 17.
